# Comparative Transcriptome Analysis of the Pacific Oyster *Crassostrea gigas* Characterized by Shell Colors: Identification of Genetic Bases Potentially Involved in Pigmentation

**DOI:** 10.1371/journal.pone.0145257

**Published:** 2015-12-22

**Authors:** Dandan Feng, Qi Li, Hong Yu, Xuelin Zhao, Lingfeng Kong

**Affiliations:** Key Laboratory of Mariculture Ministry of Education, Ocean University of China, Qingdao, China; Institute of Oceanology, Chinese Academy of Sciences, CHINA

## Abstract

**Background:**

Shell color polymorphisms of Mollusca have contributed to development of evolutionary biology and population genetics, while the genetic bases and molecular mechanisms underlying shell pigmentation are poorly understood. The Pacific oyster (*Crassostrea gigas*) is one of the most important farmed oysters worldwide. Through successive family selection, four shell color variants (white, golden, black and partially pigmented) of *C*. *gigas* have been developed. To elucidate the genetic mechanisms of shell coloration in *C*. *gigas* and facilitate the selection of elite oyster lines with desired coloration patterns, differentially expressed genes (DEGs) were identified among the four shell color variants by RNA-seq.

**Results:**

Digital gene expression generated over fifteen million reads per sample, producing expression data for 28,027 genes. A total number of 2,645 DEGs were identified from pair-wise comparisons, of which 432, 91, 43 and 39 genes specially were up-regulated in white, black, golden and partially pigmented shell of *C*. *gigas*, respectively. Three genes of *Abca1*, *Abca3* and *Abcb1* which belong to the ATP-binding cassette (ABC) transporters super-families were significantly associated with white shell formation. A *tyrosinase* transcript (CGI_10008737) represented consistent up-regulated pattern with golden coloration. We proposed that white shell variant of *C*. *gigas* could employ “endocytosis” to down-regulate *notch* level and to prevent shell pigmentation.

**Conclusion:**

This study discovered some potential shell coloration genes and related molecular mechanisms by the RNA-seq, which would provide foundational information to further study on shell coloration and assist in selective breeding in *C*. *gigas*.

## Introduction

Color polymorphisms provide tractable systems within which to examine the molecular basis of adaptation because of their often-simple patterns of inheritance [1], contributing to development of evolutionary biology and population genetics. Morgan established the modern theory of the gene based on the white-eyed *Drosophila melanogaster* [2]. Much of the pigment-based coloration in invertebrates results from products of the melanin, ommochrome, pteridine, papiliochrome and heme synthesis pathways [3]. Of them, melanin is the most widespread pigment in nature, which consists of two classes: eumelanins, which are black or brown, and pheomelanins, which are red, orange, or yellow [4]. The enzyme tyrosinase (phenol oxidase) is essential for all various melanins and even non-pigmented sclerotin [5]. And ommochromes are often found in animals that also synthesize melanins and effect on colors of yellow, orange, red, brownish and dark purple [6].

Shell color polymorphisms have always attracted interest of naturalists and biologists, but have been poorly explored in contrast to other classic polychromatism. Many molluscs are models for ecological genetics, as well as important fishery and aquaculture species. Among them, the land snail *Cepaea nemoralis* is a preeminent model for ecological genetics, because the outward color and banding phenotype is entirely genetically determined, primarily determined by a “supergene” of at least five loci [7] through classic crossing work. With shell colors being used as useful makers for selective breeding in molluscs, such as *Patinopecten yessoensis* [8], *Hyriopsis cumingii* [9], *Chlamys farreri* [10], the genetic bases and molecular mechanisms of shell color formation are receiving significantly increasing attention. Comparative transcriptome analysis of *Meretrix meretrix* suggested that *Notch*-related genes combine with calcium signaling, as an upstream component of the shell coloration determination process [11]. For *P*. *yessoensis*, the pathways of tyrosine metabolism and melanogenesis were detected in the mantle transcriptome, which might play fundamental roles in the biology of shell pigmentation [12]. Yet despite the association of melanins with some shell pigments, there exists no experimentally verified systemic molecular understanding of any shell pigmentation in any mollusc. And there is no report that genes directly responsible for shell coloration were detected and characterized. Thus the genetic bases and molecular mechanisms underlying shell pigmentation are only beginning to be elucidated.

The Pacific oyster (*Crassostrea gigas*) has the largest production among all cultured aquatic animals [13] and its coloration is of interest to the whole oyster industry [14]. Pacific oysters exhibited distinct shell colors (golden, white, and black) and the shell pigmentation has been considered as a quantitative trait that is controlled by many genes with small-effects [15]. Quantitative trait locus (QTL) for shell pigmentation and background coloration have been established with amplified fragment length polymorphism (AFLP) makers [16–17], which will facilitate our explore to the genetic mechanisms of shell coloration in the *C*. *gigas*.

**Fig 1:**
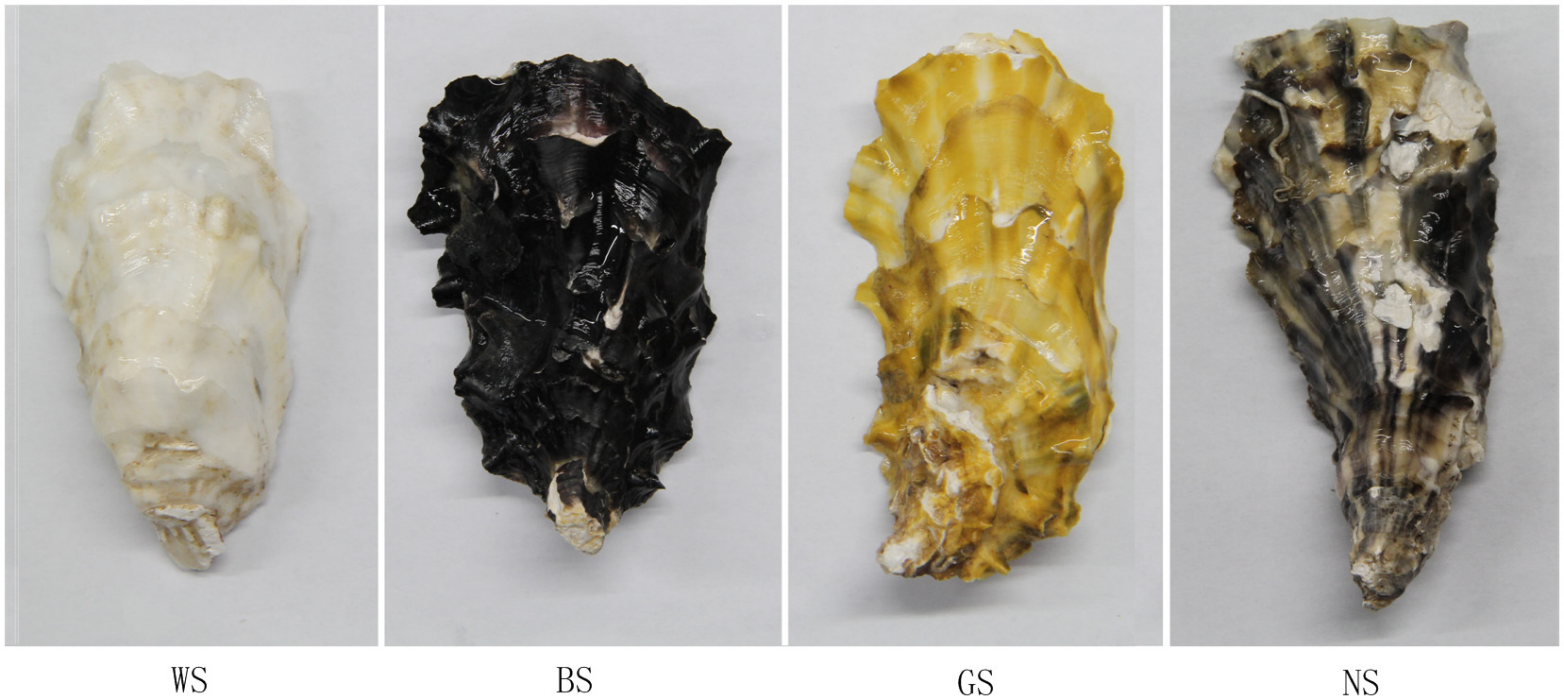
Oysters (*Crassostrea gigas*) represented four kinds of shell color variants. *WS*, the oyster of whole white shell; *BS*, the oyster of whole black shell; *GS*, the oyster of whole golden shell; *NS*, the oyster of partially pigmentation shell.

We have developed four *C*. *gigas* full-sib families characterized by shell colors, which are white shell (WS), golden shell (GS), black shell (BS) and partially pigmented shell (NS) (Fig 1), through selective breeding. These particular samples permit us to analysis specific pigmentation in shell of *C*. *gigas*. Recently, along with the rapid development and cost reduction of next generation sequencing (NGS), RNA sequencing (RNA-seq) has being a robust and comprehensive tool to analysis gene expression pattern. Moreover, a complete genome sequence of *C*. g*igas* has been accessible [18]. In this study, we used digital gene expression (DGE) profiling to investigate differentially expressed genes (DEGs) among different shell colors of *C*. g*igas*, which would provide foundational information to further study on the genetic bases and molecular mechanisms of the shell coloration.

## Materials and Methods

### Ethics statement

The Pacific oysters used in this study were cultured animals, and all the experiments on oysters were conducted according to institutional and national guidelines. No endangered or protected species was involved in the experiments of the study. No specific permission was required for the location of the culture experiment.

### Sample collections

Four kinds of Pacific oyster lines, separately named the whole white shell full-sib families (WS), whole black shell full-sib families (BS), whole golden shell full-sib families (GS), partially pigmentation shell full-sib families (NS), were developed by six-generation successive family selection and exhibited steadily hereditary shell color traits. The original parents of white, black, golden and partially pigmented *C*. *gigas* were selected locally from cultured populations in Weihai, Shandong, China. In 2014, we separately sampled offspring from the four kinds of full-sib families. We selected three oyster individuals of two-year-old from each of four kinds of full-sib families for RNA-seq, respectively. Left mantles were taken and reserved in RNA store (Dongsheng Biotech) before RNA extraction.

### RNA extraction and quality controlling

The mantle from each individual was lysed in 1 ml of Trizol Reagent (Invitrogen) for total RNA extraction according to the manufacturer’s instructions. RNA degradation and contamination was monitored on 1% agarose gels. RNA purity was checked using the NanoPhotometer^®^ spectrophotometer (Implen, CA, USA). RNA concentration was measured using Qubit^®^ RNA Assay Kit in Qubit^®^ 2.0 Flurometer (Life Technologies, CA, USA). RNA Integrity Number (RIN) was checked using the RNA Nano 6000 Assay Kit of the Bioanalyzer 2100 system (Agilent Technologies, CA, USA). At least 3 μg of total RNA was pooled from three individuals within each family, a total of four samples were used for library construction.

### Library preparation and DGE sequencing

Sequencing libraries were generated using NEBNext^®^ Ultra™ RNA Library Prep Kit for Illumina^®^ (NEB, USA) following manufacturer`s recommendations and index codes were added to attribute sequences to each sample. Library quality was assessed on the Agilent Bioanalyzer 2100 system. The quality index-coded samples were clustered on a cBot Cluster Generation System using TruSeq PE Cluster Kit v3-cBot-HS for Illumina^®^ (NEB) according to the manufacturer`s instructions. After cluster generation, the library preparations (W_ME, B_ME, G_ME and N_ME) were sequenced on an Illumina Hiseq 2500 platform (Novogene, Beijing, China) and 50 bp single-end reads were generated.

### Quality control and reads mapping

Raw data of FASTQ format (all raw tag data have been deposited in Short Read Archive (SRA) of the National Center for Biotechnology Information (NCBI)) were firstly processed through in-house Perl scripts. In this step, clean data were obtained by removing reads containing adapter, ploy-N and low quality reads from raw data. At the same time, Q20, Q30 and GC content of the clean data were calculated. All the downstream analyses were based on the clean data with high quality.

Reference genome and gene model annotation files were downloaded from genome website directly (ftp://ftp.ncbi.nlm.nih.gov/genomes/Crassostrea_gigas/). Index of the reference genome was built using Bowtie v2.0.6 [19] and single-end clean reads were aligned to the reference genome using TopHat v2.0.9 [20]. We selected TopHat as the mapping tool for that it can generate a database of splice junctions based on the gene model annotation file and thus a better mapping result than other non-splice mapping tools.

### Quantification of reads and differential expression analysis

Reads count for each gene was obtained from the mapping results by HTSeq v0.6.1 [21]. And then Reads Per Kilo base of exon model per Million mapped reads (RPKM) of each gene was calculated based on the length of the gene and reads count mapped to this gene. RPKM considers the effect of sequencing depth and gene length for the reads count at the same time, and is currently the most commonly used method for estimating gene expression levels [22].

Without strict biological replicates for each sequenced library, the read counts were adjusted by edgeR program package [23] through one scaling normalized factor. Differential expression analysis of two conditions was performed using the DEGSeq R package (1.12.0) [24]. The *P*-values were adjusted using the Benjamini and Hochberg method. Corrected *P*-value of 0.005 and log2 (fold_change) of ±1 were set as the threshold for significantly differential expression. Volcano plots were applied to intuitively show the DEGs. Hierarchical cluster analysis of DEGs union was performed to assess the transcriptional pattern variations among W_ME, B_ME, G_ME and N_ME using Cluster 3.0 [25]. Venn charts were drawn using Venny 2.0.2 (http://bioinfogp.cnb.csic.es/tools/venny/index.html) to exhibit shared or specific DEGs among different pair-wise comparisons.

### GO and KEGG enrichment analysis of DEGs

Gene ontology (GO) enrichment analysis is to identify GO terms that are significantly overrepresented in a given set of genes, which suggest possible mechanism of regulation that are put into play, or functional pathways that are activated in that condition [26]. Enrichment analysis of DEGs was implemented by the GOseq R package [27], within which gene length bias was corrected. GO terms with corrected *P*-value less than 0.05 were significantly enriched by DEGs.

Kyoto Encyclopedia of Genes and Genomes (KEGG) is a database of biological systems (http://www.genome.jp/kegg/) that integrates genomic, chemical and systemic functional information, for understanding high-level functions and utilities of the biological system, such as the cell, the organism and the ecosystem, from molecular-level information, especially large-scale molecular datasets generated by genome sequencing and other high-throughput experimental technologies [28]. The statistical enrichment of DEGs in KEGG pathways was tested by KOBAS software [29]. KEGG pathway with corrected *P*-value less than 0.05 were significantly enriched by DEGs.

### Quantitative Real-Time PCR validation

To validate the RNA-seq, 14 DEGs of interest were selected for quantitative real-time PCR (qRT-PCR) analysis. Total RNA was extracted separately from the same 12 samples used for RNA sequencing. Then cDNA was synthesized from RNA, which was used for qRT-PCR quantification, using Prime Script TM RT reagent Kit with gDNA Eraser (TaKaRa, Dalian, China). Specific primers for qRT-PCR were designed using Premier Primer 5 (S1 Table). Enlongation Factor was used as an endogenous control [30]. The amplification was performed in triplicate on the LightCycler 480 real-time PCR instrument (Roche Diagnostics, Burgess Hill, UK) using SYBR^®^ Premix Ex TaqTM (TaKaRa). Cycling parameters were 95°C for 5 min, then 40 cycles of 95°C for 5 s, 60°Cfor 20 s. Melting curve analyses were performed following amplifications to verify specific amplication. Relative gene expression data was analyzed using the comparative threshold cycle (CT) method [31]. Data were examined for homogeneity of variances (F text), and were analyzed by t test using software SPSS 13.0 with *P*<0.05.

## Results

### Sequencing and mapping of the DGE libraries

The four *C*. *gigas* cDNA libraries of different shell colors lines (WS, BS, GS, NS) were constructed and sequenced using Illumina Hiseq 2500, and the raw data were submitted to the NCBI SRA database with accession numbers of SRR1988511 (B_ME), SRR1997308 (W_ME), SRR1997309 (G_ME) and SRR1997310 (N_ME). A total of 67,101,512 clean reads filtered from 67,628,685 single-end raw reads of 50 bp produced in four libraries, with Q20 (%) varying from 98.00% to 98.11% (Table 1). As a result, the total reads length was 3.35 gigabases (Gb) for the four samples. Alignment of the sequence reads against the oyster genome yielded 84.84–85.67% of total aligned reads across the four samples, of which 66.4–69.0% fell in annotated exons, 8.8–9.8% located in introns, and the remaining 22.0–23.8% was assigned to intergenic regions (Table 1).

**Table 1:** Basic characteristic of reads in four libraries and data of sequencing reads mapping to the reference genome.

Sample name	W_ME	B_ME	G_ME	N_ME
Raw reads	16,194,954	16,180,110	18,296,565	16,957,056
Clean reads	16,062,344	16,056,566	18,127,078	16,855,524
Clean bases	0.80G	0.80G	0.91G	0.84G
Q20(%)	98.04	98.00	98.02	98.11
Q30(%)	94.39	94.31	94.34	94.53
GC content(%)	43.53	43.83	44.30	44.18
Total reads	16,062,344	16,056,566	18,127,078	16,855,524
Total mapped	13,697,765 (85.28%)	13,622,258 (84.84%)	15,528,795 (85.67%)	14,402,380 (85.45%)
Multiple mapped	2,079,192 (12.94%)	2,158,490 (13.44%)	2,528,279 (13.95%)	2,409,373 (14.29%)
Uniquely mapped	11,618,573 (72.33%)	11,463,768 (71.4%)	13,000,516 (71.72%)	11,993,007 (71.15%)
Non-splice reads	9,402,808 (58.54%)	9,227,035 (57.47%)	10,450,901 (57.65%)	9,709,764 (57.61%)
Splice reads	2,215,765 (13.79%)	2,236,733 (13.93%)	2,549,615 (14.07%)	2,283,243 (13.55%)
Exon	67.90%	68.00%	69.00%	66.40%
Intron	9.30%	9.80%	8.80%	9.80%
Intergenic	22.80%	22.20%	22.20%	23.80%

Gb: Giga base; Q20: percentage of bases with a Phred value at least 20. Q30: percentage of bases with a Phred value of at least 30.

Ratios of genes number to total gene number are presented.

The abundance of all the genes (28,027) was normalized and calculated by RPKMs method using uniquely mapped reads. Genes with RPKMs in the interval 0–1 were considered not to be expressed or to be presented at very low levels; genes with RPKMs over 60 were considered to be expressed at a very high level. The distributions of the expression levels of all the genes were similar among all four libraries (S2 Table). About 66% of the total number of genes (28,027) were expressed (RPKM≥1), and more than 1,962 genes were highly expressed (RPKM>60) in each library (S2 Table).

### Analysis of differentially expressed genes

**Fig 2:**
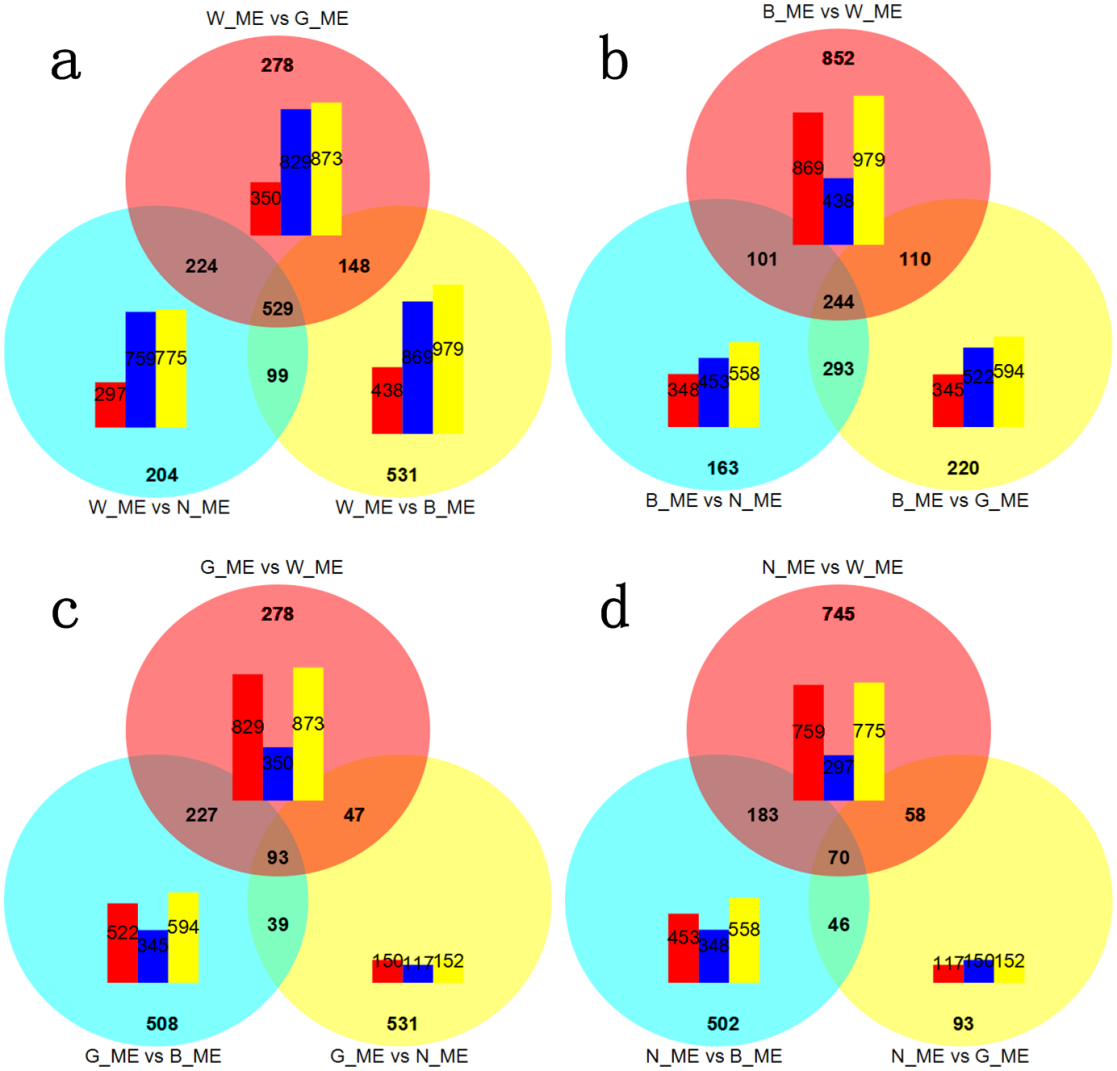
Venn digrams and histograms for numbers comparisons of DEGs among four DGE libraries (W_ME, B_ME, G_ME, N_ME). Venn digrams represented the shared or specific DEGs among three pair-wise comparison between some one DGE library with any one of the three. The numbers of the shared DEGs are in the cross area, while the numbers of the specific DGEs are in the single area. Histogram represented the numbers of down-regulated (red), up-regulated (blue), and annotation (yellow) genes of DEGs in every pair-wise comparison. (a), W_ME compared with other three libraries in pairs (b), B_ME compared with other three libraries in pairs (c), G_ME compared with other three libraries in pairs (d), N_ME compared with other three libraries in pairs.

To minimize the false-positive DEGs among the four libraries, the read counts were adjusted by the edgeR program with one scaling normalized factor. The DEGs in pairs among four libraries were detected by DEGseq with corrected *P*-value <0.005 & |log2 (fold_change)| > 1. Consequently, a total of 2,645 DEGs were detected, of which 474, 158, 56, 48 genes specially were up-regulated in WS, BS, GS and NS respectively. A total number of 1307, 1179, 1056 DEGs were respectively detected from the comparison of W_ME vs B_ME, W_ME vs G_ME, W_ME vs N_ME, which were significantly larger than the others—867, 801, 267, respectively from the comparison of B_ME vs G_ME, B_ME vs N_ME, G_ME vs N_ME. A total number of 869, 829, 759 up-regulated genes were detected respectively from the comparison of W_ME vs B_ME, W_ME vs G_ME, W_ME vs N_ME, which were significantly larger than the down-regulated genes—438, 350, 297. The numbers of shared or specific DEGs among three pair-wise comparisons between some DGE library with any of the other three were represented by Venn digrams (Fig 2). And the numbers of down-regulated, up-regulated and annotation genes of DEGs in every pair-wise comparison were represented by Histogram (Fig 2). That visually displayed the significant expansion of DEGs and up-regulated genes in comparison between W_ME with any of other three. Consistent results were globally exhibited in volcano plots and hierarchical clustering. Volcano plots visually displayed that there were more DEGs in pair-wise comparisons relating to white variant, compared to those in the other pair-wise comparisons, and that the up-regulated genes dominated the DGEs in every comparison when white variant compared with any of other three variants (S1 Fig). Hierarchical clustering exhibited that the whole expression of DEGs in WS was distinguishable from that in other three samples (BS, GS, NS), and every variant has itself special high expression genes clusters (S2 Fig).

### Enrichment analysis of DEGs

**Fig 3:**
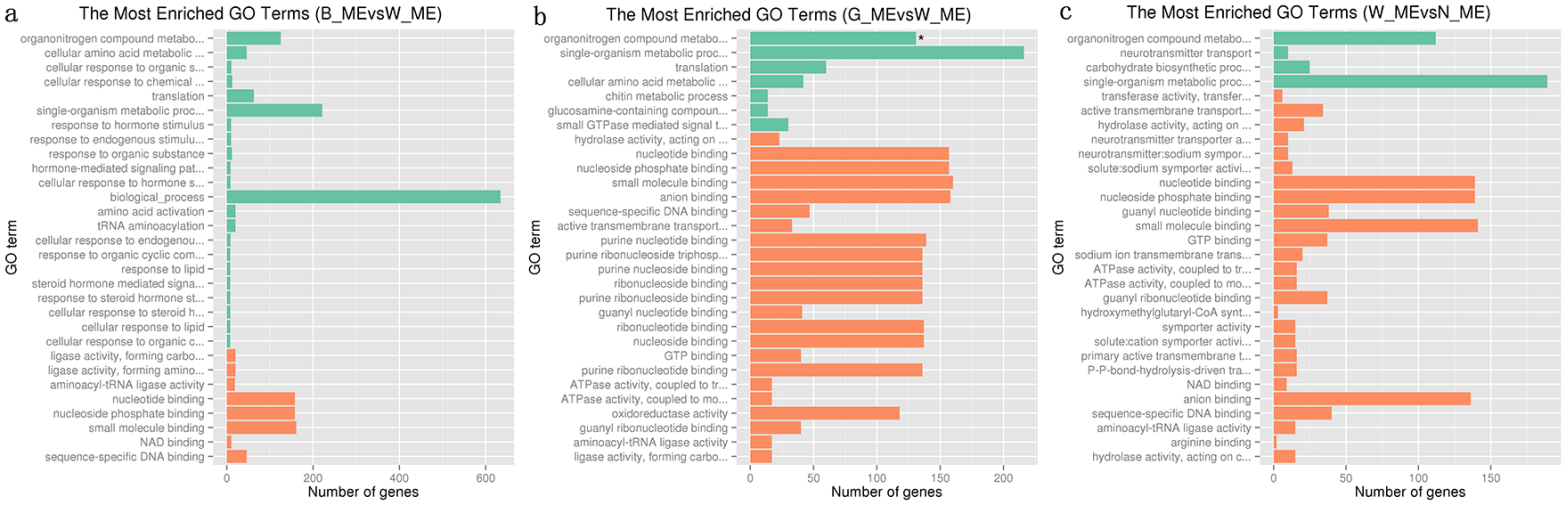
Gene ontology classification of up-regulated genes in comparisons between WS with any of other three variants. Orange represented molecular function, green represented biological process.

We performed GO enrichment analysis to identify the main molecular function of these DEGs in each pair-wise comparison among four variants. When WS compared with any of other three variants, the most significantly enriched GO terms based on up-regulated DEGs were shared by three GO terms of “nucleotide binding”, “small molecule binding”, “nucleoside phosphate binding” (Fig 3), showing uniformly consistent. The three GO terms consist of the same 90 up-regulated genes shared by the three pair-wise comparisons (S3 Table), except that “small molecule binding” includes more two genes. When BS compared with any of other three variants, the most significantly enriched GO term based on up-regulated DEGs was only one GO term of “calcium ion binding” (S3 Fig), consisting of 19 up-regulated genes (S4 Table). When NS or GS compared with the others, there was no GO term showing consistent.

**Fig 4:**
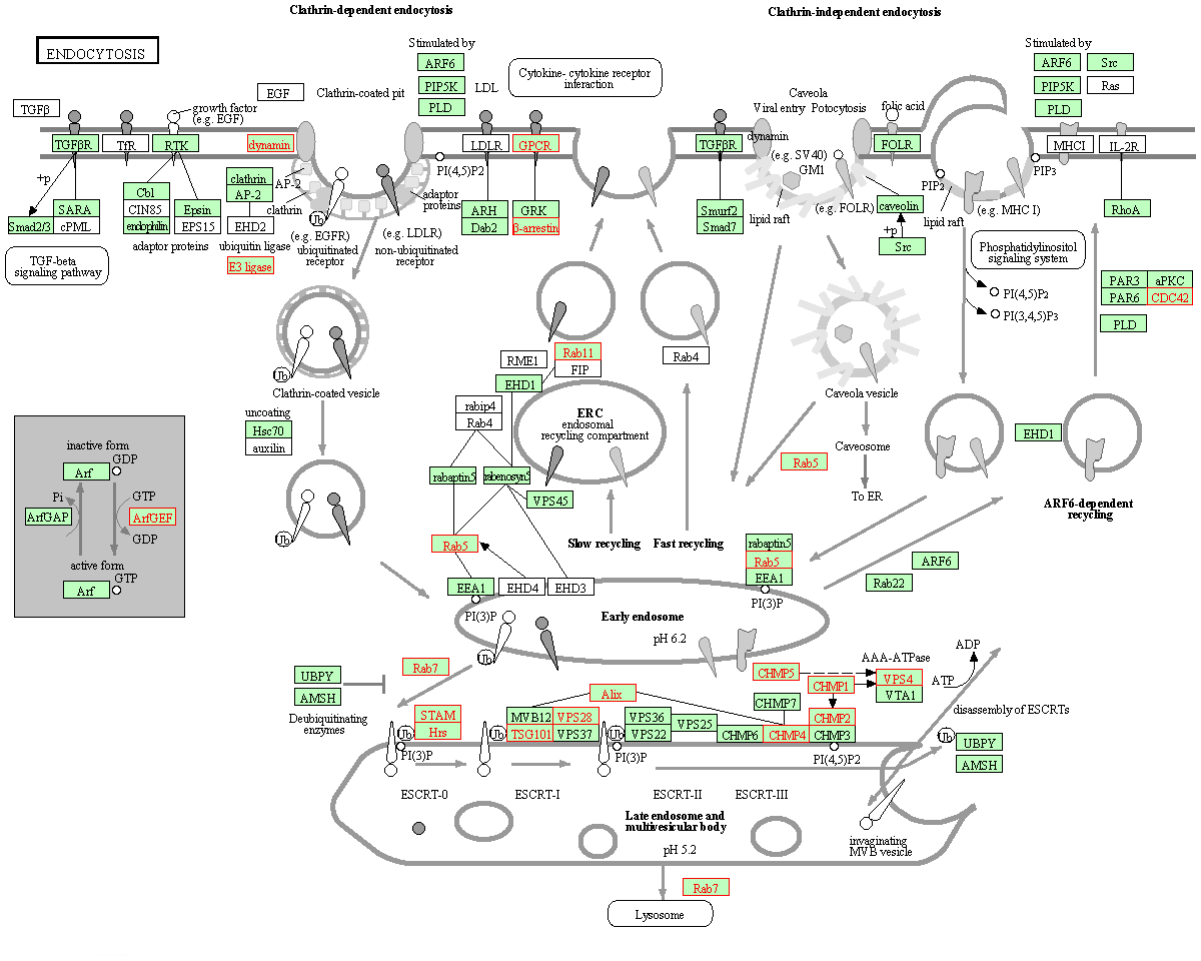
The endocytosis pathway was significantly enriched from the KEGG pathways and shared based on DEGs in comparisons of WS vs any of other three variants. Genes with green background were discovered in the RNA-seq, and genes with red text were up-regulated in WS.

We performed KEGG enrichment analysis to further discover the metabolic processes and signal transduction pathways, which were related with shell color polymorphisms. When WS compared with other three variants, “endocytosis” was consistently and significantly enriched among three comparisons with corrected *P*-Value <0.05 (Fig 4). The other three libraries had no shared significantly enrichment pathway.

Furthermore, we conducted function enrichment analysis using DEGs specially expressed in WS, considering WS had more DEGs compared with other variants. And three GO terms of “nucleotide binding”, “small molecule binding”, “nucleoside phosphate binding” (S4 Fig) and one KEGG pathway of “Endocytosis” with corrected *P*-Value of 0.00026 <0.05 were most significantly enriched, which showed consistent with the aforementioned shared function enrichment analysis based on up-regulated DEGs when WS compared with any of other three variants.

### Identification of genes with special interest

Tyrosinase is essential for the synthesis of widespread and various melanins. Tyrosinase gene family has undergone large expansions in bivalves and suggests an important function in tanning periostracum proteins [32]. 27 *tyrosinase* gene family members have been identified in *C*. *gigas* [18]. We analysed the 27 tyrosinase related genes and identified 12 transcripts coding for *tyrosinase* related genes (Table 2) from DEGs. Of them, the group of WS vs BS of down-regulation has the least differentially expressed *tyrosinase* related gene with none; the group of BS vs GS of up-regulation has the most differentially expressed *tyrosinase* genes with six. And the gene *Tyro_pinma* (CGI_10008737) was up-regulated only when GS or NS compared with WS or BS.

**Table 2:** *Tyrosinase* transcripts in every pair-wise comparison.

Group		Transcripts of *tyrosinase* list					
WS vs BS	up-regulation	CGI_10026230	CGI_10009318	CGI_10011916	CGI_10017214		
WS vs BS	down-regulation	none					
WS vs GS	up-regulation	CGI_10017983	CGI_10009318	CGI_10009319	CGI_10007793	CGI_10017214	
WS vs GS	down-regulation	CGI_10008737					
WS vs NS	up-regulation	CGI_10009318	CGI_10011916	CGI_10017214			
WS vs NS	down-regulation	CGI_10008737	CGI_10012743				
BS vs GS	up-regulation	CGI_10017983	CGI_10009319	CGI_10011911	CGI_10007753	CGI_10021076	CGI_10021075
BS vs GS	down-regulation	CGI_10008737	CGI_10026230	CGI_10011916	CGI_10012743		
BS vs NS	up-regulation	CGI_10009319	CGI_10021076				
BS vs NS	down-regulation	CGI_10008737	CGI_10026230	CGI_10012743			
GS vs NS	up-regulation	CGI_10011916					
GS vs NS	down-regulation	CGI_10017983					

To gain further insights into the association of the DEGs with shell coloration, we integrated the DEGs and previously reported QTLs by comparing their scaffold positions with those of the QTLs. A total number of 16 DEGs were located in the same scaffold with QTLs. The positions of the 16 genes on the scaffold and detailed information about the QTLs were shown in S6 Table. Among the 16 genes, one was found to be located within the QTL region that was predicted to have significant genetic effect on shell background color.

### Validation by qRT-PCR

Fourteen genes of interest were selected to perform qRT-PCR and compare with the RNA-seq data by gene’s transcript level (S7 Table). The results showed that the expression patterns of *Rb11a*, *Abca1*, *Abca3*, *Rab7a*, *Notch2-human*, *Tsg101*, *Notch2-rat*, *Dyi3*, *Tyr1*, *Efcb5* and *Scp* agreed well between RNA-seq and qRT-PCR. Three genes of *Notch*, *Tyr-3*, *Pif* showed a similar trend in change of expression pattern between RNA-seq and qRT-PCR, which showed that qRT-PCR was more sensitive for detection of DEGs.

## Discussion

Shell color polymorphisms have promoted development of evolutionary biology and population genetics, but have been poorly understood in contrast to other classic polychromatism. With the progressing in using the marker of shell colors to breed for shellfish varieties of definite phenotype and good character, the genetic bases and molecular mechanisms of shell color formation are receiving significantly increasing attention. This study is designed to discover the genes related with the shell color polymorphisms and molecular mechanisms of shell pigmentation regulation by comparative transcriptome analysis among different shell color variants of *C*. *gigas*.

### WS had more DEGs compared with other variants

This study applied DGE analysis to identify the genes, which influence shell color variants formation. Specifically, DEGs that are promising to regulate the shell coloration of *C*. *gigas* were identified by comparing the gene expression profiles in the mantle among four kinds of shell color variants. All expression level results including density distribution of expression, hierarchical clustering, volcano plots and Venn charts indicated that WS was distinguishable from the other three variants (BS, GS and NS). Interestingly, WS with no pigmentation had the most DEGs, especially up-regulated genes, than BS, GS and NS with more or less pigmentation. All four libraries were constructed from oyster strains obtained by artificial selection in successive generations, eliminating the interference of genetic background difference between wild and farmed strains. That phenomenon was not an accident but the result of white comparing with other colors, which exist in sheep [33], pearl mussel [9], clam [11], and scallop [34]. Thus, the white variant is regulated by special molecular mechanism involved in more genes compared with other color variants. And function enrichment based on DEGs specially expressed in WS showed that the white variant was mainly regulated by endocytosis involved in genes in terms of “nucleotide binding”, “small molecule binding”, “nucleoside phosphate binding”.

### Analysis of molecular mechanisms on non-pigment shell formation

When WS compared with other three variants, three shared GO terms of “nucleotide binding”, “small molecule binding”, “nucleoside phosphate binding”, were most significantly enriched. These GO terms included in the same 91 up-regulated genes, except that “small molecule binding” included more two genes. Among them, the genes of *Abca1*, *Abca3*, *Abcb1*, fully named as ATP-binding cassette sub-family A member 1, ATP-binding cassette sub-family A member 3 and multidrug resistance protein 1, respectively, suggested associated with white coloration. These three genes belong to the ATP-binding cassette (ABC) transporters super-families, which is one of the largest transporter families and is present in all kingdoms of life [35]. *D*. *melanogaster white* and its thologues in insect are probably the most thoroughly characterized ABC transporters and are involved in the uptake of pigment precursors in the developing eye [35], which determined the coloration of the compound eye. Mutations located at various sites in the alleles of *white* differently influenced on the transportation of pigment precursors. Mutations identified within the nucleotide binding domain caused substantial and similar decreases of red and brown pigments. ABC transporters that effected on coloration have also been discovered in silkworm. *Bmwh3* was responsible for the transportation of ommochrome precursors and uric acid into pigment granules and urate granules, respectively [36]. Null mutants of *Bmwh3* have white eyes and eggs because they lack ommochrome pigments [37] and have the distinct phenotype of translucent larval skin because the lack of uric acid [36]. Similarly, we can hypothesis that there were some ABC transporters responsible for transportation of pigment related substrate to influence pigmentation in oyster. *Abca1* and *Abcg1*, the human homology of the *Drosophila white* gene [38], synergize to mediate cholesterol export in peripheral tissues [39]. And cholesterol regulates melanogenesis and increases melanin content in human epidermal melanocytes and melanoma cells in a concentration-dependent manner [40]. Furthermore, *Abcg1* in WS was also found to express more than in BS with log2 (fold_change) of 1.0249. So we supposed that *Abca1* and *Abcg1* synergize to mediate cholesterol export in mantle of *C*.*gigas* to decrease melanin. *Abca3* was expressed in human choroid-retinal pigment epithelium and retinal pigment epithelial cells, mutations in which were associated with cataract-microcornea syndrome (CCMC) [41]. However, the exact mechanism of *Abca3* action and its role in dominant CCMC pathogenesis remain unclear, and future functional studies will be important [41]. *Abcb1* have been identified as potential unconjugated bilirubin transporters, which export the pigment from the cells [42]. And *Abcb1* was also found to function in melanoma cells, but the specific role of *Abcb1* in the melanomas remains to be established [43]. Above all, the three ABC genes- *Abca1*, *Abca3*, *Abcb1*, especially *Abca1* showed promising influence on non-pigment shell formation.

When WS compared with other three variants, “endocytosis” was significantly enriched among three comparisons by KEGG analysis. Endocytosis is a mechanism for cells to remove ligands, nutrients, plasma membrane proteins and lipids from the cell surface, bringing them into the cell interior [44]. And endocytosis influences pigment granules formation. For instance, the aforementioned *white*, *scarlet* and *brown* genes are located in the membranes of pigment granules within pigment cells and retinula cells, and correlate to pigment granule function through endocytosis [45]. Specifically, ligands and receptors that mediate cell-cell interactions during development were removed from the cell surface by endocytosis [46]. Subsequently, many of these internalized proteins were detected in multivesicular bodies (MVBs), and some of them were delivered to lysosomes or pigment granules in *D*. *melanogaster* and melanosomes in mammalian cells by trafficking through MVBs [47]. As shown in the endocytosis pathway figure, most up-regulated genes aggregated in the ESCRT pathway of endocytosis, within which components are highly conserved in yeast, insects and humans [48]. The ESCRT machinery consists of the peripheral membrane protein complexes ESCRT-0, -I, -II, -III, and Vps4–Vta1, and the ALIX homodimer, the majority of which are from the class E vacuolar protein sorting (VPS) morphological group [49]. Mutants in Class E VPS result vacuoles larger than wild-type, with a very large, aberrant late endosome/MVB or prevacuolar compartment [50], which is similar with the *deep orange (dor)* mutant in *D*. *melanogaster*. The complete loss of *dor* function resulted in giant multivesicular structures in mutant pigment cells and white patches in adult compound eyes [51]. The *dor* and *carnation* form a fly homolog of the HOPS-complex, which are required for cargo delivery, not only to lysosomes, but also to their pigment-containing relatives [46]. Moreover, ESCRT-I and perhaps additional ESCRT components are required to ensure tyrosinase-related protein1 (*Tyrp1*) steady-state localization to the mature melanosome limiting membrane [52]. Melanosomes are tissue-specific lysosome-related organelles of pigment cells, in which melanins are synthesized, stored and secreted [53]. *Tyrp1* trafficking was blocked in cells in which ESCRT-I component *Tsg101* is depleted or in which ESCRT function is generally inhibited by dominant-negative overexpression of inactive *Vps4* or of *Tsg101* [52]. *Hrs*, a component of the “ESCRT-0” complex, is enriched in the clathrin-containing coats of stage I melanosomes [53]. *Hrs* over-expression disrupted the early endosomal trafficking and depleted *Tyrp1* from mature melanosomes [52]. Besides these components of the ESCRT pathway, *Rab5*, *Rab7* and *Rab11* have also been found in the endocytosis as up-regulated genes. These three genes belong to a large family Rab GTPases in eukaryotic genomes, which has been identified as central regulators of intracellular transport [54]. *Rab5*, *Rab7* and *Rab11* occupy distinct membrane domains sequentially traversed by recycling cargo. And cargo destined for degradation is first internalized into *Rab5* domains on early endosomes and later appears in *Rab7* domains on late endosomes [55]. Moreover, *Rab7* is associated with melanosome biogenesis and the transport of *Tyrp1* from the trans-Golgi network to melanosomes [56], just like *Hrs* [52]. Altogether, the endocytosis pathway is composed of distinct organelles and subdomains, some of which are closely associated with the biogenesis of pigment granules and largely influence pigmentation. The identified up-regulated genes belonging to class E VPS or Rab GTPases in the endocytosis are promising to effect on shell coloration.

### Analysis of mechanisms on pigmented shell formation

When BS compared with other three variants, the most significantly enriched and shared GO term is only “calcium ion binding”, which is consistent with transcriptome analysis of shell color-related genes in *M*. *meretrix*. And *Notch*-related genes in this term are predicted to combine with calcium signaling, as an upstream component of the shell coloration determination process, which are involved in the shell pigmentation and color patterning [11]. *Notch* signaling pathway functions in an enormous diversity of developmental processes, and has a simple framework that is highly conserved throughout the animal kingdom [57]. It is an essential cell-cell interaction mechanism, which regulates processes such as cell proliferation, cell fate decisions, differentiation or stem cell maintenance [58]. Signaling is triggered by binding of the ligands *Delta* or *Serrate*, resulting in a concerted two-step proteolysis of the *Notch* receptor [46]. Both the *Notch* receptor and its ligands, *Delta* and *Serrate*, are transmembrane proteins with large extracellular domains that consist primarily of epidermal growth factor (EGF)-like repeats [57]. Though the basic core *Notch*-transduction pathway is same in most *Notch*-dependent processes, the mechanisms that regulate the pathway are different and still unclear [57]. It is noteworthy that the endocytosis and endosomal sorting of the receptor effect on the notch signaling pathway. Studies have shown that *Notch* colocalizes with the small Rab GTPases *Rab5* and *Rab7*, which are both markers of the endocytic pathway [59]. Mutations in the ESCRT components *VPS25* and *TSG101* result in dramatic hyperplasia that is due to over-activation of the notch pathway [60–61]. In the *D*. *melanogaster* retina, *notch* is down-regulated by endocytosis, which is essential for pigment cell determination and survival [62]. In this study, *Notch* was presented in the aforementioned “calcium ion binding”, and *Notch2* was shared by the three groups down-regulated genes when WS compared with other three variants. Taken together, white shell oysters could employ endocytosis to down-regulate *notch* level and lead to melanoblasts apoptosis, preventing pigmentation.

When the other two variants (GS, NS) compared with the WS and BS, there were no significantly shared GO terms or KEGG pathways discovered. It is interpreted that the molecular mechanism that control golden shell and partially pigmentation shell is the intermediate of the throughout molecular pathway controlling from whole-pigmentation to non-pigmentation in shell, just like pigment metabolism in *D*. *melanogaster*. The synthesis of all pigments begin with the conversion of tyrosine to dopa, some of which are then converted to black dopa melanin, while some of which are then converted to pigment precursors dopamine [5]. Dopamine can be converted to brown melanin or other pigment precursors, N-β-alanyl dopamine (NBAD, yellow sclerotin) and N-acetyl dopamine (NADA, colorless or transparent sclerotin) [5]. Thus, every comparison between one of GS or NS with other one represents different part of the throughout pigmentation molecular pathway, which share relatively less similarity. Even though, all comparisons represent valuable information on shell coloration and still remain to discover when more information on shell coloration mechanisms is available.

### Analysis of genes with special interest

Melanin, as the most widespread pigments in nature, has also been found in molluscan [63]. Dopa melanin (black), dopamine melanin (brown), NBAD (yellowish tan) and NADA (unpigmented) are all synthesized by the catalysis of tyrosinase (phenol oxidases) [5]. Tyrosinase-like protein existed in the biosynthesis of both melanin and betalain (nitrogen-containing water-soluble compounds, yellow/red colors) [34]. Thus tyrosinase is essential for varied coloration. We identified 12 transcripts coding for *tyrosinase* related genes from DEGs, of which none was identified in the group of BS vs WS of up-regulation. In contrast, the group of BS vs GS of up-regulation has the most differentially expressed *tyrosinase* genes with six. That may suggest that *tyrosinase* is essential for golden shell formation in oysters. It was noteworthy that the gene *Tyro* (CGI_10008737) was up-regulated only when GS or NS compared with WS or BS. In the other way, *Tyro* (CGI_10008737) was up-regulated only when shell represented yellow coloration and was promising to influence yellow coloration.

Integrated analysis of DEGs and QTLs revealed that 16 DEGs located in the same scaffold with QTLs, significantly suggested their effect on pigmentation or shell background coloration. Of them, one (CGI_10014768) located in the region of AFLP maker (O11f375) linked with background color, which could be special to *C*.*gigas* and regulate the shell coloration without annotation. Dynein intermediate chain 3 (*Dyi3*) is a component of dynein, which are molecular motors responsible for many different types of microtubule-based motility [64]. And it has been suggested to power melanosome transport and be responsible for pigment motility in the cell [65]. Protein PIF (*Pif*) has been demonstrated to regulate nacre formation [66], and its function in shell coloration needed further study. The other linked DEGs represented no clue to pigmentation and needed more research.

In conclusion, we performed RNA-seq for *C*. *gigas* from different shell colors full-sib families, which were developed by successive family selection. And a total number of 2,645 DEGs were identified from pair-wise comparisons, of which 432, 91, 43, 39 genes were specially up-regulated in WS, BS, GS and NS respectively, which provided invaluable RNA-seq data to assist in identifying genetic bases of shell coloration. The GO terms of “nucleotide binding”, “small molecule binding”, “nucleoside phosphate binding”, “calcium ion binding” and KEGG pathway of “endocytosis” provided significant clues to understand the molecular mechanisms of shell color polymorphisms and to identify potential shell coloration genes. We proposed that white shell variant could employ “endocytosis” to down-regulate *notch* level and to prevent pigmentation. Three genes of *Abca1*, *Abca3* and *Abcb1* which belong to the ABC transporters super-families were potentially associated with white shell formation. A *tyrosinase* transcript (CGI_10008737) represented consistent up-regulated pattern with golden coloration. This study represents the first attempt to identify the genetic bases and molecular mechanisms underlying shell coloration in transcriptome scale, and provides fundamental information for systematic analysis of shell coloration and assist in selective breeding in *C*. *gigas*.

## Supporting Information

S1 Fig Volcano plots displayed differentially expressed genes in each pair-wise comparison. (TIF)

S2 Fig Hierarchical clustering of DGEs union in four DGEs libraries (W_ME, B_ME, G_ME, and N_ME), using the RNA-seq data based on log_10_RPKM value. (TIFF)

S3 Fig Gene Ontology classification based on up-regulated genes in comparisons between BS vs any of other three variants. (TIF)

S4 Fig Gene Ontology classification based on DEGs specially expressed in WS. (TIF)

S1 Table Primer sequences for qRT-PCR. (DOCX)

S2 Table Distribution of gene expressions in the four shell colors oysters. (DOCX)

S3 Table The shared DEGs among the GO terms of nucleotide binding, small molecule binding, nucleoside phosphate binding based on up-regulated genes in W_ME. (DOCX)

S4 Table The shared DEGs among the GO term of calcium ion binding based on up-regulated genes in B_ME. (DOCX)

S5 Table The shared DEGs among the KEGG pathways of endocytosis based on up-regulated genes in W_ME. (DOCX)

S6 Table Detailed information on the DEGs potentially linked with the reported QTLs for shell pigmentation and background coloration. (DOCX)

S7 Table qRT-PCR validation and comparative analyses with RNA-seq data. (DOCX)
