# Supplementary figures and images for: Comparative Transcriptome Analysis of the Pacific Oyster Crassostrea gigas Characterized by Shell Colors: Identification of Genetic Bases Potentially Involved in Pigmentation
Source: PLoS One. 2015 Dec 22;10(12):e0145257. doi: 10.1371/journal.pone.0145257 (PMC4691203; doi:10.1371/journal.pone.0145257)

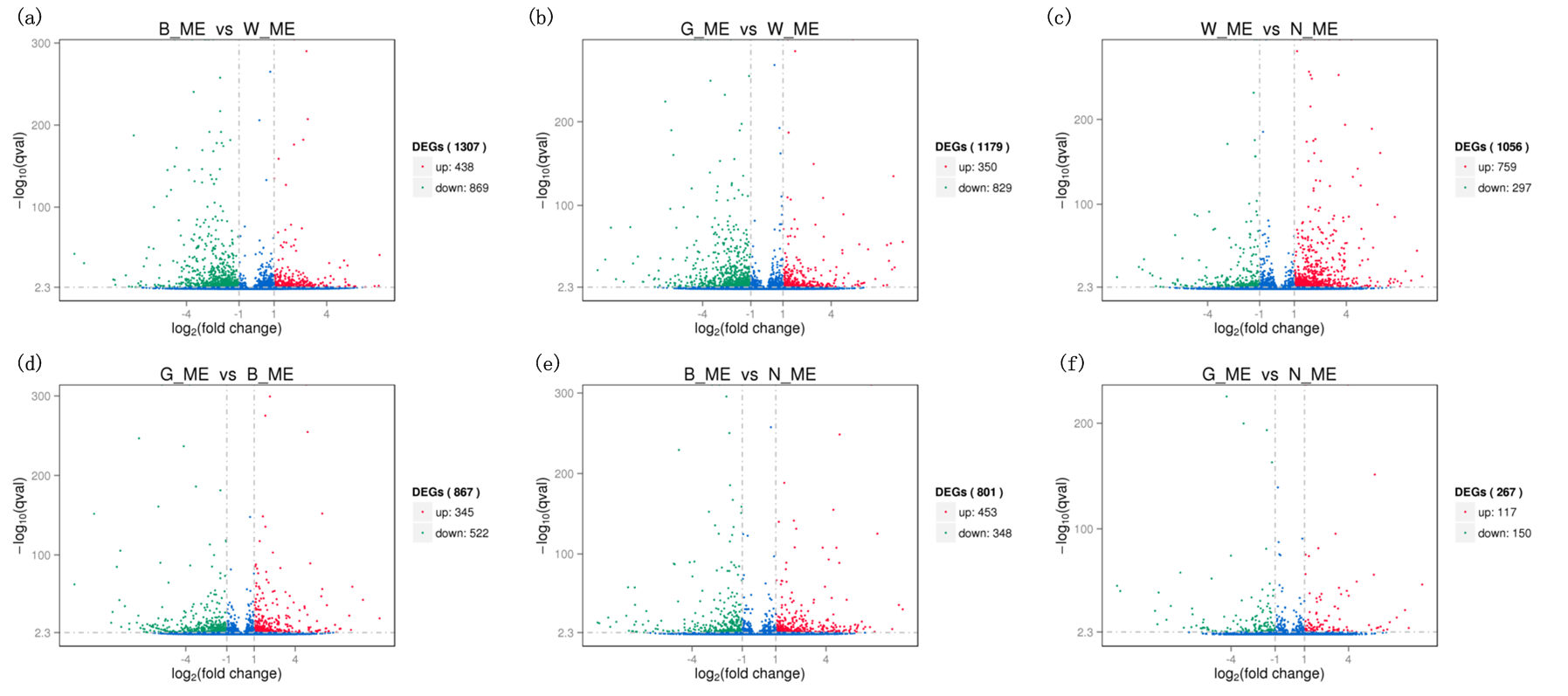

Supplement: S1 Fig — (TIF) [file pone.0145257.s001.tif]

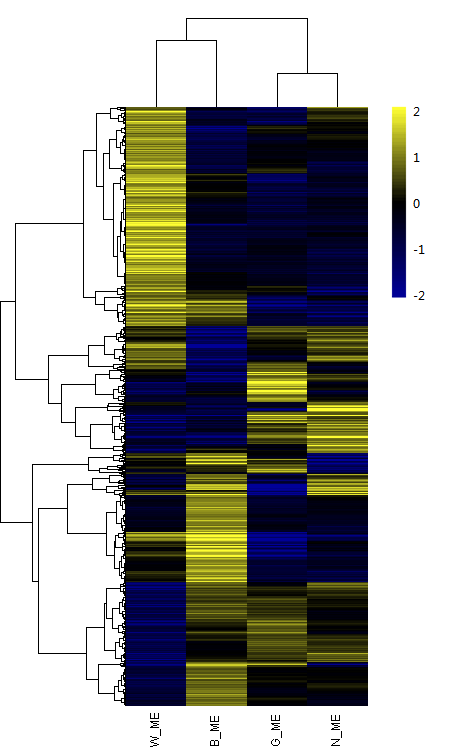

Supplement: S2 Fig — (TIFF) [file pone.0145257.s002.tiff]

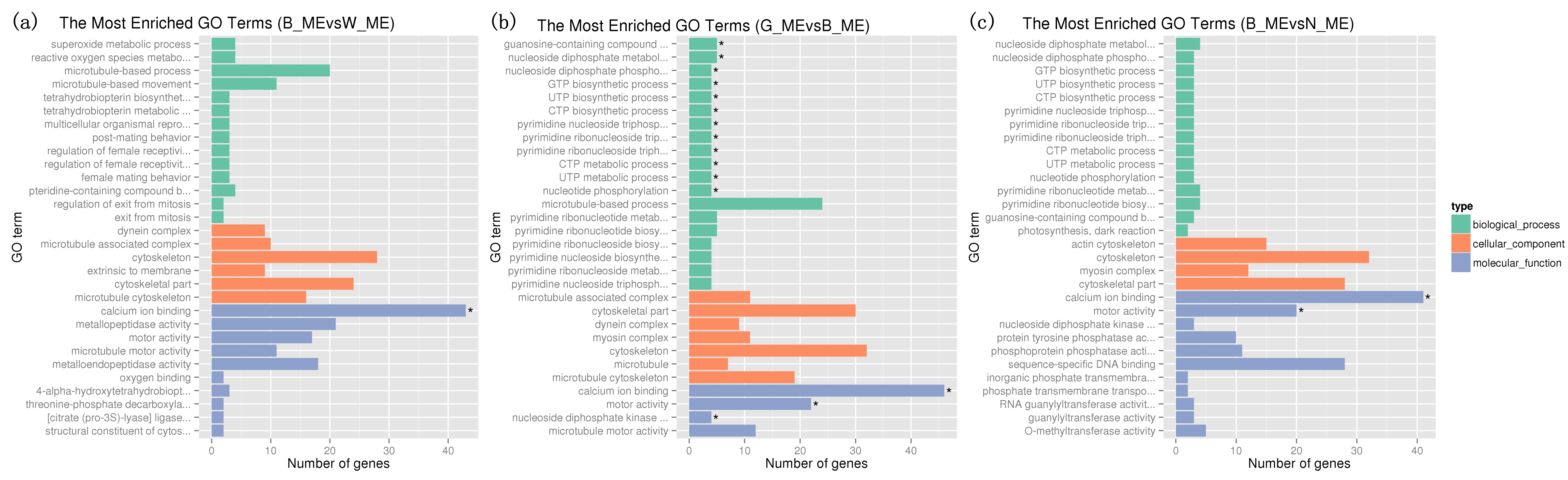

Supplement: S3 Fig — (TIF) [file pone.0145257.s003.tif]

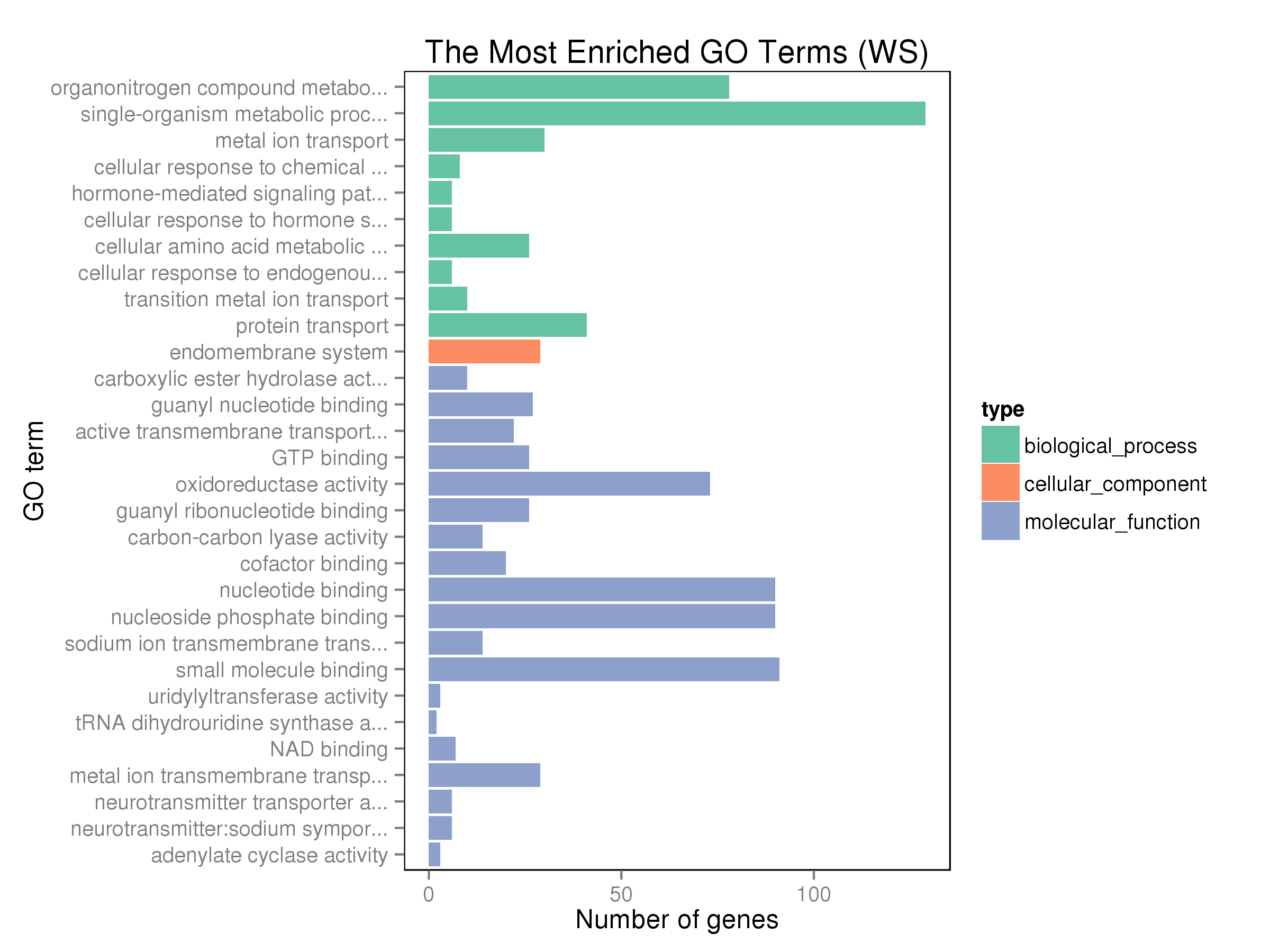

Supplement: S4 Fig — (TIF) [file pone.0145257.s004.tif]
