# Supplementary material for: Comparative Transcriptome Analysis of the Pacific Oyster Crassostrea gigas Characterized by Shell Colors: Identification of Genetic Bases Potentially Involved in Pigmentation
Source: PLoS One. 2015 Dec 22;10(12):e0145257. doi: 10.1371/journal.pone.0145257 (PMC4691203; doi:10.1371/journal.pone.0145257)
Supplement: S1 Table — (DOCX) [file pone.0145257.s005.docx]

**S1 Table Primer sequences for qRT-PCR**

| **Gene Name** | **GenBank** | **Primer Sequences** |
| --- | --- | --- |
| *Rb11a* | CGI_10005650 | F ACCAATCTTCTGTCCAGGTTCAC |
|  |  | R GCCTTCAATGTCTTTCCGTCT |
| *Abca1* | CGI_10020425 | F ATGTAGGTCAGGTCACGGTTCT |
|  |  | R CCTTGCCATGAGTCGGTTG |
| *Abca3* | CGI_10021664 | F CAAGGCAAGGTTCACCGAGTT |
|  |  | R CTGGAACCGACCCACATTT |
| *Efcb5* | CGI_10008411 | F CGACCACAGTGGAATTGGGT |
|  |  | R CATCTGCGAGGGTTTCGTAC |
| *Scp* | CGI_10006806 | F CTTCAGGGCGTTTGGTCAC |
|  |  | R TGGGAACTTTATCATTGGATGC |
| *Notch* | CGI_10013186 | F CTGCTTTGCTGGTGCCTGT |
|  |  | R GAGCGAGGACCCACATAACC |
| Rab7a | CGI_10001648 | F AATAGAGCGGTGACTGCTAAGAG |
|  |  | R TAGCGACAGTTTGGAAAGCCT |
| Notch2  -human | CGI_10007010 | F ATCTGTGGTTCAAACGCTAAGTG |
|  |  | R ACAAGTCCCAAACGCATCC |
| Tyr-3 | CGI_10009318 | F TCACCCAAGCCAGCAGATTAT |
|  |  | R TTGTACCACCCGTCGTAGTTAAG |
| Pif | CGI_10012353 | F ACGCCAACTGTCCTTATG |
|  |  | R TGATCCAACCTACACTCCC |
| Tsg101 | CGI_10028835 | F ATGACCCAGTTCAAAGATTTACG |
|  |  | R GGTGCCATCCAAGTTCAGAAG |
| Notch2-rat | CGI_10004966 | F AACATCCCAACTCCGTCACC |
|  |  | R CGCTTCTGCTACATTGCCAAC |
| Dyi3 | CGI_10019307 | F CATGGTAGGATGTGAACAAGGCT |
|  |  | R AATGGATTGCGTTGAAGTGAGTAG |
| Tyr1 | CGI_10009319 | F TGTTTGTAGCTTATGTTGCCGTG |
|  |  | R TGATGGGCGACAGACTCTACC |

F: Forward primer; R: Reverse primer.
