# Supplementary material for: Comparative Transcriptome Analysis of the Pacific Oyster Crassostrea gigas Characterized by Shell Colors: Identification of Genetic Bases Potentially Involved in Pigmentation
Source: PLoS One. 2015 Dec 22;10(12):e0145257. doi: 10.1371/journal.pone.0145257 (PMC4691203; doi:10.1371/journal.pone.0145257)
Supplement: S2 Table — (DOCX) [file pone.0145257.s006.docx]

**S2 Table Distribution of gene expressions in the four shell colors oysters**

| RPKM Interval | B_ME | W_ME | G_ME | N_ME |
| --- | --- | --- | --- | --- |
| 0~1 | 9,029 | 9,858 | 9,836 | 9,542 |
|  | (32.22%) | (35.17%) | (35.09%) | (34.05%) |
| 1~3 | 3,278 | 3,912 | 3,637 | 3,708 |
|  | (11.70%) | (13.96%) | (12.98%) | (13.23%) |
| 3~15 | 8,034 | 7,583 | 7,697 | 7,925 |
|  | (28.67%) | (27.06%) | (27.46%) | (28.28%) |
| 15~60 | 5,518 | 4,515 | 4,816 | 4,856 |
|  | (19.69%) | (16.11%) | (17.18%) | (17.33%) |
| >60 | 2,168 | 2,159 | 2,041 | 1,996 |
|  | (7.74%) | (7.70%) | (7.28%) | (7.12%) |

RPKM: Reads per kilo bases per million reads.

Ratios of genes number to total gene number are presented in parentheses.
