# Supplementary material for: Comparative Transcriptome Analysis of the Pacific Oyster Crassostrea gigas Characterized by Shell Colors: Identification of Genetic Bases Potentially Involved in Pigmentation
Source: PLoS One. 2015 Dec 22;10(12):e0145257. doi: 10.1371/journal.pone.0145257 (PMC4691203; doi:10.1371/journal.pone.0145257)
Supplement: S3 Table — (DOCX) [file pone.0145257.s007.docx]

| **S3 Table The shared DEGs among the GO terms of nucleotide binding, small molecule binding , nucleoside phosphate binding based on up-regulated genes in W_ME** | |
| --- | --- |
| Gene_id | Blast swiss prot |
| CGI_10000886 | sp\|P08183\|MDR1_HUMAN Multidrug resistance protein 1 OS=*Homo sapiens* GN=ABCB1 |
| CGI_10001203 | sp\|P70705\|ATP7A_RAT Copper-transporting ATPase 1 OS=*Rattus norvegicus* GN=Atp7a |
| CGI_10001553 | sp\|Q4P331\|IF4A_USTMA ATP-dependent RNA helicase eIF4A OS=*Ustilago maydis* (strain 521 / FGSC 9021) GN=TIF1 |
| CGI_10002841 | sp\|Q60803\|TRAF3_MOUSE TNF receptor-associated factor 3 OS=*Mus musculus* GN=Traf3 |
| CGI_10003627 | sp\|Q8NFT2\|STEA2_HUMAN Metalloreductase STEAP2 OS=*Homo sapiens* GN=STEAP2 |
| CGI_10003945 | sp\|Q75J93\|CPAS1_DICDI Circularly permutated Ras protein 1 OS=*Dictyostelium discoideum* GN=cpras1 |
| CGI_10003999 | sp\|Q6TGS6\|SYYC_DANRE Tyrosine--tRNA ligase, cytoplasmic OS=*Danio rerio* GN=yars |
| CGI_10004396 | sp\|P16435\|NCPR_HUMAN NADPH--cytochrome P450 reductase OS=*Homo sapiens* GN=POR |
| CGI_10005254 | sp\|Q2KJG3\|SYNC_BOVIN Asparagine--tRNA ligase, cytoplasmic OS=*Bos taurus* GN=NARS |
| CGI_10005261 | sp\|Q4QQW8\|PLBL2_RAT Putative phospholipase B-like 2 OS=*Rattus norvegicus* GN=Plbd2 |
| CGI_10005650 | sp\|Q5ZJN2\|RB11A_CHICK Ras-related protein Rab-11A OS=*Gallus gallus* GN=RAB11A |
| CGI_10006103 | sp\|O95363\|SYFM_HUMAN Phenylalanine--tRNA ligase, mitochondrial OS=*Homo sapiens* GN=FARS2 |
| CGI_10006412 | sp\|Q5ZHZ0\|DX39B_CHICK Spliceosome RNA helicase DDX39B OS=*Gallus gallus* GN=DDX39B |
| CGI_10006511 | sp\|Q61035\|SYHC_MOUSE Histidine--tRNA ligase, cytoplasmic OS=*Mus musculus* GN=Hars |
| CGI_10007005 | sp\|P62510\|ERR3_RAT Estrogen-related receptor gamma OS=*Rattus norvegicus* GN=Esrrg |
| CGI_10007664 | sp\|Q7ZVK3\|SIR2_DANRE NAD-dependent protein deacetylase sirtuin-2 OS=*Danio rerio* GN=sirt2 |
| CGI_10008099 | sp\|P46461\|NSF1_DROME Vesicle-fusing ATPase 1 OS=*Drosophila melanogaster* GN=comt |
| CGI_10008386 | sp\|Q9CXF4\|TBC15_MOUSE TBC1 domain family member 15 OS=*Mus musculus* GN=Tbc1d15 |
| CGI_10009438 | sp\|Q8WXR4\|MYO3B_HUMAN Myosin-IIIb OS=*Homo sapiens* GN=MYO3B |
| CGI_10009641 | sp\|P49696\|SYVC_TAKRU Valine--tRNA ligase OS=*Takifugu rubripes* GN=vars |
| CGI_10009840 | sp\|Q9NV88\|INT9_HUMAN Integrator complex subunit 9 OS=*Homo sapiens* GN=INTS9 |
| CGI_10009856 | sp\|P55043\|RAD_RAT GTP-binding protein RAD OS=*Rattus norvegicus* GN=Rrad |
| CGI_10009859 | sp\|Q99MN1\|SYK_MOUSE Lysine--tRNA ligase OS=*Mus musculus* GN=Kars |
| CGI_10010298 | sp\|Q29HY3\|CDC42_DROPS Cdc42 homolog OS=*Drosophila pseudoobscura* GN=Cdc42 |
| CGI_10010381 | sp\|Q566C8\|ANR54_RAT Ankyrin repeat domain-containing protein 54 OS=*Rattus norvegicus* GN=Ankrd54 |
| CGI_10010676 | sp\|O89017\|LGMN_MOUSE Legumain OS=*Mus musculus* GN=Lgmn |
| CGI_10012229 | sp\|O70496\|CLCN7_MOUSE H(+)/Cl(-) exchange transporter 7 OS=*Mus musculus* GN=Clcn7 |
| CGI_10012376 | sp\|P21575\|DYN1_RAT Dynamin-1 OS=*Rattus norvegicus* GN=Dnm1 |
| CGI_10012977 | sp\|Q9XZV3\|GNAO_GEOCY Guanine nucleotide-binding protein G(o) subunit alpha OS=*Geodia cydonium* |
| CGI_10013631 | sp\|Q13637\|RAB32_HUMAN Ras-related protein Rab-32 OS=*Homo sapiens* GN=RAB32 |
| CGI_10013657 | sp\|P70705\|ATP7A_RAT Copper-transporting ATPase 1 OS=*Rattus norvegicus* GN=Atp7a |
| CGI_10014151 | sp\|P38606\|VATA_HUMAN V-type proton ATPase catalytic subunit A OS=*Homo sapiens* GN=ATP6V1A |
| CGI_10014381 | sp\|O76050\|NEU1A_HUMAN Neuralized-like protein 1A OS=*Homo sapiens* GN=NEURL |
| CGI_10014578 | sp\|Q8CHQ0\|FBX4_MOUSE F-box only protein 4 OS=*Mus musculus* GN=Fbxo4 |
| CGI_10014915 | sp\|P05153\|PCKGC_CHICK Phosphoenolpyruvate carboxykinase, cytosolic [GTP] OS=*Gallus gallus* GN=PCK1 |
| CGI_10014971 | sp\|Q7PD79\|GNAS_ANOGA Guanine nucleotide-binding protein G(s) subunit alpha OS=*Anopheles gambiae* GN=G-s-alpha-60A |
| CGI_10015583 | sp\|O14638\|ENPP3_HUMAN Ectonucleotide pyrophosphatase/phosphodiesterase family member 3 OS=*Homo sapiens* GN=ENPP3 |
| CGI_10015668 | sp\|P97363\|SPTC2_MOUSE Serine palmitoyltransferase 2 OS=*Mus musculus* GN=Sptlc2 |
| CGI_10015799 | sp\|Q32LQ6\|MFSD1_DANRE Major facilitator superfamily domain-containing protein 1 OS=*Danio rerio* GN=mfsd1 |
| CGI_10015931 | sp\|Q06396\|ARF1_ORYSJ ADP-ribosylation factor 1 OS=*Oryza sativa* GN=Os01g0813400 |
| CGI_10016438 | sp\|O75886\|STAM2_HUMAN Signal transducing adapter molecule 2 OS=*Homo sapiens* GN=STAM2 |
| CGI_10016663 | sp\|Q9Y2I7\|FYV1_HUMAN 1-phosphatidylinositol 3-phosphate 5-kinase OS=*Homo sapiens* GN=PIKFYVE |
| CGI_10016690 | sp\|G5E8K5\|ANK3_MOUSE Ankyrin-3 OS=*Mus musculus* GN=Ank3 |
| CGI_10016812 | sp\|Q9R0M6\|RAB9A_MOUSE Ras-related protein Rab-9A OS=*Mus musculus* GN=Rab9a |
| CGI_10016837 | sp\|Q6P8C8\|ARL8A_XENTR ADP-ribosylation factor-like protein 8A OS=*Xenopus tropicalis* GN=arl8a |
| CGI_10017042 | sp\|Q9NFT7\|HXK2_DROME Hexokinase type 2 OS=*Drosophila melanogaster* GN=Hex-t2 |
| CGI_10017084 | sp\|Q641C9\|CD123_XENLA Cell division cycle protein 123 homolog OS=*Xenopus laevis* GN=cdc123 |
| CGI_10017721 | sp\|F1Q4S1\|ATP9B_DANRE Probable phospholipid-transporting ATPase IIB OS=*Danio rerio* GN=atp9b |
| CGI_10017999 | sp\|P08183\|MDR1_HUMAN Multidrug resistance protein 1 OS=*Homo sapiens* GN=ABCB1 |
| CGI_10018112 | sp\|Q03042\|KGP1_DROME cGMP-dependent protein kinase, isozyme 1 OS=*Drosophila melanogaster* GN=Pkg21D |
| CGI_10018499 | sp\|P31409\|VATB_DROME V-type proton ATPase subunit B OS=*Drosophila melanogaster* GN=Vha55 |
| CGI_10019126 | sp\|Q9NFT7\|HXK2_DROME Hexokinase type 2 OS=*Drosophila melanogaster* GN=Hex-t2 |
| CGI_10019132 | sp\|Q99973\|TEP1_HUMAN Telomerase protein component 1 OS=*Homo sapiens* GN=TEP1 |
| CGI_10019552 | sp\|P12276\|FAS_CHICK Fatty acid synthase OS=*Gallus gallus* GN=FASN |
| CGI_10020425 | sp\|P41233\|ABCA1_MOUSE ATP-binding cassette sub-family A member 1 OS=*Mus musculus* GN=Abca1 |
| CGI_10020580 | sp\|P35295\|RAB20_MOUSE Ras-related protein Rab-20 OS=*Mus musculus* GN=Rab20 |
| CGI_10020638 | sp\|Q9Z1G4\|VPP1_MOUSE V-type proton ATPase 116 kDa subunit a isoform 1 OS=*Mus musculus* GN=Atp6v0a1 |
| CGI_10020639 | sp\|Q8C2P3\|DUS1L_MOUSE tRNA-dihydrouridine(16/17) synthase [NAD(P)(+)]-like OS=*Mus musculus* GN=Dus1l |
| CGI_10020770 | sp\|P21575\|DYN1_RAT Dynamin-1 OS=*Rattus norvegicus* GN=Dnm1 |
| CGI_10020906 | sp\|B8ARK7\|SIR1_ORYSI NAD-dependent protein deacetylase SRT1 OS=*Oryza sativa* GN=SRT1 |
| CGI_10020907 | sp\|Q9FE17\|SIR1_ARATH NAD-dependent protein deacetylase SRT1 OS=*Arabidopsis thaliana* GN=SRT1 |
| CGI_10021664 | sp\|Q99758\|ABCA3_HUMAN ATP-binding cassette sub-family A member 3 OS=*Homo sapiens* GN=ABCA3 |
| CGI_10021957 | sp\|Q3SYZ4\|SYDC_BOVIN Aspartate--tRNA ligase, cytoplasmic OS=*Bos taurus* GN=DARS |
| CGI_10022111 | sp\|P67999\|KS6B1_RAT Ribosomal protein S6 kinase beta-1 OS=*Rattus norvegicus* GN=Rps6kb1 |
| CGI_10023357 | sp\|Q9LVX0\|ARI3_ARATH Probable E3 ubiquitin-protein ligase ARI3 OS=*Arabidopsis thaliana* GN=ARI3 |
| CGI_10023430 | sp\|A4IHT0\|FIGL1_XENTR Fidgetin-like protein 1 OS=*Xenopus tropicalis* GN=fignl1 |
| CGI_10023625 | sp\|P11717\|MPRI_HUMAN Cation-independent mannose-6-phosphate receptor OS=*Homo sapiens* GN=IGF2R |
| CGI_10023685 | sp\|O73853\|CP17A_ICTPU Steroid 17-alpha-hydroxylase/17,20 lyase OS=*Ictalurus punctatus* GN=cyp17a1 |
| CGI_10024093 | sp\|P86854\|PLCL_MYTGA Perlucin-like protein OS=*Mytilus galloprovincialis* |
| CGI_10024370 | sp\|Q9BX10\|GTPB2_HUMAN GTP-binding protein 2 OS=*Homo sapiens* GN=GTPBP2 |
| CGI_10024608 | sp\|Q7ZWS1\|DUS3L_XENLA tRNA-dihydrouridine(47) synthase [NAD(P)(+)]-like OS=*Xenopus laevis* GN=dus3l |
| CGI_10025300 | sp\|Q9BW19\|KIFC1_HUMAN Kinesin-like protein KIFC1 OS=*Homo sapiens* GN=KIFC1 |
| CGI_10025593 | sp\|P97432\|NBR1_MOUSE Next to BRCA1 gene 1 protein OS=*Mus musculus* GN=Nbr1 |
| CGI_10025807 | sp\|O08582\|GTPB1_MOUSE GTP-binding protein 1 OS=*Mus musculus* GN=Gtpbp1 |
| CGI_10026281 | sp\|O43490\|PROM1_HUMAN Prominin-1 OS=*Homo sapiens* GN=PROM1 |
| CGI_10026353 | sp\|Q864R9\|MRP1_MACFA Multidrug resistance-associated protein 1 OS=*Macaca fascicularis* GN=ABCC1 |
| CGI_10027132 | sp\|Q5ZHW4\|RAB5B_CHICK Ras-related protein Rab-5B OS=*Gallus gallus* GN=RAB5B |
| CGI_10027758 | sp\|Q3KQW7\|LIMC1_XENLA LIM and calponin homology domains-containing protein 1 OS=*Xenopus laevis* GN=limch1 |
| CGI_10027842 | sp\|P27674\|GTR1_BOVIN Solute carrier family 2, facilitated glucose transporter member 1 OS=*Bos taurus* GN=SLC2A1 |
| CGI_10028436 | sp\|P38024\|PUR6_CHICK Multifunctional protein ADE2 OS=*Gallus gallus* GN=AIRC |
| CGI_10028449 | sp\|Q0VA42\|F188B_XENTR Protein FAM188B OS=*Xenopus tropicalis* GN=fam188b |
| CGI_10028856 | sp\|P49588\|SYAC_HUMAN Alanine--tRNA ligase, cytoplasmic OS=*Homo sapiens* GN=AARS |
| CGI_10028931 | sp\|Q9NXL6\|SIDT1_HUMAN SID1 transmembrane family member 1 OS=*Homo sapiens* GN=SIDT1 |

The listed DEGs were those have Swiss Prot description.
