# Supplementary material for: Comparative Transcriptome Analysis of the Pacific Oyster Crassostrea gigas Characterized by Shell Colors: Identification of Genetic Bases Potentially Involved in Pigmentation
Source: PLoS One. 2015 Dec 22;10(12):e0145257. doi: 10.1371/journal.pone.0145257 (PMC4691203; doi:10.1371/journal.pone.0145257)
Supplement: S4 Table — (DOCX) [file pone.0145257.s008.docx]

| **S4 Table The shared DEGs among the GO term of calcium ion binding based on up-regulated genes in B_ME** | |
| --- | --- |
| Gene ID | Blast swiss prot |
| CGI_10005202 | sp\|Q6V0I7\|FAT4_HUMAN Protocadherin Fat 4 OS=*Homo sapiens* GN=FAT4 |
| CGI_10006133 | sp\|Q75N90\|FBN3_HUMAN Fibrillin-3 OS=*Homo sapiens* GN=FBN3 |
| CGI_10006806 | sp\|P02637\|SCP_MIZYE Sarcoplasmic calcium-binding protein OS=*Mizuhopecten yessoensis* |
| CGI_10007658 | sp\|Q96RW7\|HMCN1_HUMAN Hemicentin-1 OS=*Homo sapiens* GN=HMCN1 |
| CGI_10008411 | sp\|A4FU69\|EFCB5_HUMAN EF-hand calcium-binding domain-containing protein 5 OS=*Homo sapiens* GN=EFCAB5 |
| CGI_10011293 | sp\|Q95NR9\|CALM_METSE Calmodulin OS=*Metridium senile* |
| CGI_10011703 | sp\|Q0VAA2\|CN16B_HUMAN Uncharacterized protein C14orf166B OS=*Homo sapiens* GN=C14orf166B |
| CGI_10013186 | sp\|P07207\|NOTCH_DROME Neurogenic locus Notch protein OS=*Drosophila melanogaster* GN=N |
| CGI_10013342 | sp\|P10079\|FBP1_STRPU Fibropellin-1 OS=*Strongylocentrotus purpuratus* GN=EGF1 |
| CGI_10014601 | sp\|Q5THR3\|EFCB6_HUMAN EF-hand calcium-binding domain-containing protein 6 OS=*Homo sapiens* GN=EFCAB6 |
| CGI_10015609 | sp\|Q9Y6R7\|FCGBP_HUMAN IgGFc-binding protein OS=*Homo sapiens* GN=FCGBP |
| CGI_10016406 | sp\|Q32LE3\|CETN1_BOVIN Centrin-1 OS=*Bos taurus* GN=CETN1 |
| CGI_10017611 | sp\|Q9D2J7\|ANKR5_MOUSE Ankyrin repeat domain-containing protein 5 OS=*Mus musculus* GN=Ankrd5 |
| CGI_10017672 | sp\|Q9ULI3\|HEG1_HUMAN Protein HEG homolog 1 OS=*Homo sapiens* GN=HEG1 |
| CGI_10023898 | sp\|P02599\|CALM_DICDI Calmodulin OS=*Dictyostelium discoideum* GN=calA |
| CGI_10025417 | sp\|P42325\|NCAH_DROME Neurocalcin homolog OS=*Drosophila melanogaster* GN=Nca |
| The listed DEGs were those have Swiss Prot description. | |
