# Supplementary material for: Comparative Transcriptome Analysis of the Pacific Oyster Crassostrea gigas Characterized by Shell Colors: Identification of Genetic Bases Potentially Involved in Pigmentation
Source: PLoS One. 2015 Dec 22;10(12):e0145257. doi: 10.1371/journal.pone.0145257 (PMC4691203; doi:10.1371/journal.pone.0145257)
Supplement: S5 Table — (DOCX) [file pone.0145257.s009.docx]

| **S5 Table The shared DEGs among the KEGG pathways of endocytosis based on up-regulated genes in W_ME** | |
| --- | --- |
| Gene_id | Blast swiss prot |
| CGI_10000381 | sp\|Q5XGW6\|CHM4B_XENLA Charged multivesicular body protein 4b OS=*Xenopus laevis* GN=chmp4b |
| CGI_10002841 | sp\|Q60803\|TRAF3_MOUSE TNF receptor-associated factor 3 OS=*Mus musculus* GN=Traf3 |
| CGI_10005242 | sp\|Q8R0S2\|IQEC1_MOUSE IQ motif and SEC7 domain-containing protein 1 OS=*Mus musculus* GN=Iqsec1 |
| CGI_10005650 | sp\|Q5ZJN2\|RB11A_CHICK Ras-related protein Rab-11A OS=*Gallus gallus* GN=RAB11A |
| CGI_10007579 | sp\|Q7ZW25\|CHM2A_DANRE Charged multivesicular body protein 2a OS=*Danio rerio* GN=chmp2a |
| CGI_10008208 | sp\|Q5ZKX1\|CHM1B_CHICK Charged multivesicular body protein 1b OS=*Gallus gallus* GN=CHMP1B |
| CGI_10010298 | sp\|Q29HY3\|CDC42_DROPS Cdc42 homolog OS=*Drosophila pseudoobscura pseudoobscura* GN=Cdc42 |
| CGI_10010435 | sp\|Q0VD48\|VPS4B_BOVIN Vacuolar protein sorting-associated protein 4B OS=*Bos taurus* GN=VPS4B |
| CGI_10012376 | sp\|P21575\|DYN1_RAT Dynamin-1 OS=*Rattus norvegicus* GN=Dnm1 |
| CGI_10013051 | sp\|Q9WU78\|PDC6I_MOUSE Programmed cell death 6-interacting protein OS=*Mus musculus* GN=Pdcd6ip |
| CGI_10014480 | sp\|Q0V8S0\|HGS_BOVIN Hepatocyte growth factor-regulated tyrosine kinase substrate OS=*Bos taurus* GN=HGS |
| CGI_10016438 | sp\|O75886\|STAM2_HUMAN Signal transducing adapter molecule 2 OS=*Homo sapiens* GN=STAM2 |
| CGI_10016812 | sp\|Q9R0M6\|RAB9A_MOUSE Ras-related protein Rab-9A OS=*Mus musculus* GN=Rab9a |
| CGI_10017761 | sp\|Q4R562\|ARRB1_MACFA Beta-arrestin-1 OS=*Macaca fascicularis* GN=ARRB1 |
| CGI_10020770 | sp\|P21575\|DYN1_RAT Dynamin-1 OS=*Rattus norvegicus* GN=Dnm1 |
| CGI_10024176 | sp\|Q9VCZ3\|OCTB1_DROME Octopamine receptor beta-1R OS=*Drosophila melanogaster* GN=oa2 |
| CGI_10025617 | sp\|Q7T339\|CHMP5_DANRE Charged multivesicular body protein 5 OS=*Danio rerio* GN=chmp5 |
| CGI_10027132 | sp\|Q5ZHW4\|RAB5B_CHICK Ras-related protein Rab-5B OS=*Gallus gallus* GN=RAB5B |
| CGI_10028266 | sp\|Q6NVL7\|CHM2B_XENTR Charged multivesicular body protein 2b OS=*Xenopus tropicalis* GN=chmp2b |
| CGI_10028835 | sp\|Q99816\|TS101_HUMAN Tumor susceptibility gene 101 protein OS=*Homo sapiens* GN=TSG101 |
