# Supplementary material for: Comparative Transcriptome Analysis of the Pacific Oyster Crassostrea gigas Characterized by Shell Colors: Identification of Genetic Bases Potentially Involved in Pigmentation
Source: PLoS One. 2015 Dec 22;10(12):e0145257. doi: 10.1371/journal.pone.0145257 (PMC4691203; doi:10.1371/journal.pone.0145257)
Supplement: S6 Table — (DOCX) [file pone.0145257.s010.docx]

**S6 Table Detailed information on the DEGs potentially linked with the reported QTLs for shell pigmentation and background coloration.**

| **Gene_id** | **Annotation** | **Position (bp)** | **Position (scaffold)** | **Previously reported QTL** | | | |
| --- | --- | --- | --- | --- | --- | --- | --- |
|  |  |  |  | **Maker** | **Marker location (bp)** | **Trait** | **References** |
| CGI_10012345 | DLEC1_HUMAN | 10050-36500 | scaffold43786 | B7f360 | 239517-239875 | Pigmentation | Ge et al. 2014 |
| CGI_10012347 | None | 42980-48205 | scaffold43786 | B7f360 | 239517-239875 | Pigmentation | Ge et al. 2014 |
| CGI_10012348 | HMCN1_HUMAN | 57956-132417 | scaffold43786 | B7f360 | 239517-239875 | Pigmentation | Ge et al. 2014 |
| CGI_10012352 | PIF_PINMG | 181917-199788 | scaffold43786 | B7f360 | 239517-239875 | Pigmentation | Ge et al. 2014 |
| CGI_10012353 | PIF_PINMG | 209050-214923 | scaffold43786 | B7f360 | 239517-239875 | Pigmentation | Ge et al. 2014 |
| CGI_10025861 | CRFR2_XENLA | 7962-27483 | scaffold733 | A4f275 | 1375-1620 | Pigmentation | Ge et al. 2014 |
| CGI_10025870 | CJ112_HUMAN | 240072-273270 | scaffold733 | A4f275 | 1375-1620 | Pigmentation | Ge et al. 2014 |
| CGI_10025876 | BP10_PARLI | 408619-548638 | scaffold733 | A4f275 | 1375-1620 | Pigmentation | Ge et al. 2014 |
| CGI_10025887 | CES1D_RAT | 740608-745472 | scaffold733 | A4f275 | 1375-1620 | Pigmentation | Ge et al. 2014 |
| CGI_10025889 | MEGF6_HUMAN | 766241-772186 | scaffold733 | A4f275 | 1375-1620 | Pigmentation | Ge et al. 2014 |
| CGI_10014767 | RL23A_RAT | 270297-274575 | scaffold542 | O11f375 | 322329-322682 | Ground color | Ge et al. 2015 |
| CGI_10014768 | None | 314748-329563 | scaffold542 | O11f375 | 322329-322682 | Ground color | Ge et al. 2015 |
| CGI_10019290 | GUN_MYTED | 113010-122923 | scaffold363 | I7f200 | 198652-198828 | Ground color | Ge et al. 2015 |
| CGI_10019305 | None | 418126-419400 | scaffold363 | I7f200 | 198652-198828 | Ground color | Ge et al. 2015 |
| CGI_10019307 | DYI3_HELCR | 461207-464146 | scaffold363 | I7f200 | 198652-198828 | Ground color | Ge et al. 2015 |
| CGI_10020119 | WIN1_SCHPO | 527435-541725 | scaffold461 | I7f270 | 567185-567428 | Ground color | Ge et al. 2015 |

Maker: The amplified fragment length polymorphism (AFLP) maker was used for the detection of QTLs.
