# Supplementary material for: Comparative Transcriptome Analysis of the Pacific Oyster Crassostrea gigas Characterized by Shell Colors: Identification of Genetic Bases Potentially Involved in Pigmentation
Source: PLoS One. 2015 Dec 22;10(12):e0145257. doi: 10.1371/journal.pone.0145257 (PMC4691203; doi:10.1371/journal.pone.0145257)
Supplement: S7 Table — (DOCX) [file pone.0145257.s011.docx]

**S7 Table qRT-PCR validation and comparative analyses with RNA-seq data**

| **Gene** | **qRT-PCR** | | | | **qRT-PCR** | | | **RNA-seq** | | |
| --- | --- | --- | --- | --- | --- | --- | --- | --- | --- | --- |
|  | △CT (mean±S.E.) | | | | the expression ratio 2^-△△CT^ | | | log_2_(fold_change) | | |
|  |  |  |  |  | (*t*-test for equality of means) | | |  | | |
|  | WS | BS | GS | NS | WS vs BS | WSvs GS | WS vs NS | WS vs BS | WS vs GS | WS vs NS |
| *Rb11a* | 7.53±0.70 | 11.01±0.49 | 12.72±0.51 | 12.57±1.15 | 12.65* | 40.69** | 25.52* | 6.27** | 6.62** | 3.88** |
|  |  |  |  |  | (*P*=0.019) | (*P*=0.005) | (*P*=0.028) |  |  |  |
| *Abca3* | 11.46±1.67 | 15.89±0.82 | 14.82±1.46 | 12.97±2.41 | 38.44* | 12.53 | 5.83 | 3.37** | 3.06** | 3.25** |
|  |  |  |  |  | (*P*=0.009) | (*P*=0.083) | (*P*=0.184) |  |  |  |
| *Abca1* | 7.18±0.47 | 10.85±0.61 | 9.10±0.71 | 8.14±0.32 | 12.15* | 3.29 | 2.08 | 3.43** | 2.53** | 1.78** |
|  |  |  |  |  | (*P*=0.010) | (*P*=0.097) | (*P*=0.179) |  |  |  |
| Rab7a | 5.22±0.56 | 5.93±0.04 | 6.64±0.07 | 6.49±0.19 | 1.93* | 2.78* | 2.79 | 1.63** | 1.76** | 2.05** |
|  |  |  |  |  | (*P*=0.021) | (*P*=0.022) | (*P*=0.075) |  |  |  |
| Notch2  -human | 12.71±1.84 | 8.21±0.22 | 9.57±0.29 | 9.09±0.06 | 0.1049* | 0.2632* | 0.1969* | -2.67** | -2.29** | -2.83** |
|  |  |  |  |  | (*P*=0.025) | (*P*=0.030) | (*P*=0.018) |  |  |  |
| Tyr-3 | 6.83±0.94 | 8.17±0.53 | 8.70±0.47 | 8.41±0.73 | 2.60 | 3.77 | 3.13 | 1.34** | 1.26** | 1.51** |
|  |  |  |  |  | (*P*=0.933) | (*P*=0.874) | (*P*=0.745) |  |  |  |
| Pif | 5.20±0.57 | 6.79±0.43 | 6.74±0.42 | 6.29±0.09 | 3.26 | 3.31 | 2.49 | 2.05** | 1.25** | 1.11 ** |
|  |  |  |  |  | (*P*=0.606) | (*P*=0.171) | (*P*=0.061) |  |  |  |
| Tsg101 | 8.1O±0.06 | 8.67±0.017 | 9.67±0.07 | 9.35±0.27 | 1.49 | 2.97 * | 2.32* | 1.37** | 1.61** | 1.43** |
|  |  |  |  |  | (*P*=0.058) | (*P*=0.007) | (*P*=0.046) |  |  |  |
|  |  |  |  |  | BS vs WS | BS vs GS | BS vsNS | BS vs WS | BS vs GS | BS vs NS |
| *Efcb5* | 11.80±0.58 | 9.22±0.37 | 11.56±0.48 | 11.01±0.03 | 5.57* | 4.90* | 3.68* | 1.09** | 1.42** | 1.35** |
|  |  |  |  |  | (*P*=0.027) | (*P*=0.020) | (*P*=0.040) |  |  |  |
| *Scp* | 12.46±1.15 | 7.02±0.12 | 13.45±1.33 | 13.82±1.09 | 23.64* | 41.84* | 65.57* | 2.85** | 6.07** | 3.89** |
|  |  |  |  |  | (*P*=0.040) | (*P*=0.039 ) | (*P*=0.024) |  |  |  |
| Notch | 8.45±0.41 | 7.21±0.39 | 7.81±0.82 | 8.33±0.31 | 2.34 | 1.19 | 2.22 | 1.23** | 1.16** | 1.34** |
|  |  |  |  |  | (P=0.095 ) | (P=0.564 ) | (P=0.094) |  |  |  |
| Notch2-rat | 14.27±0.69 | 10.72±0.06 | 12.06±0.03 | 11.61±0.01 | 9.71* | 2.53 | 1.36* | 2.10**** | 1.69 **** | 1.44 **** |
|  |  |  |  |  | (P=0.022 ) | (P=0.123 ) | (P=0.026) |  |  |  |
| Dyi3 | 10.28±1.11 | 8.18±0.05 | 10.39±0.13 | 10.48±0.13 | 2.83* | 4.58 | 4.90 | 0.79**** | 1.33 **** | 1.22 **** |
|  |  |  |  |  | (P=0.020 ) | (P=0.132 ) | (P=0.093) |  |  |  |
| Tyr1 | 9.09±0.35 | 7.83±0.01 | 11.23±0.25 | 10.26±0.14 | 2.25 | 5.34 | 10.24* | *1.00*** | *3.50*** | *1.99*** |
|  | |  |  |  | (P=0.074 ) | (P=0.088) | (P=0.027) |  |  |  |

**, P<0.005; * , P<0.05.
